# Cost and Effectiveness of Using Facebook Advertising to Recruit Young Women for Research: PREFER (Contraceptive Preferences Study) Experience

**DOI:** 10.2196/15869

**Published:** 2019-11-29

**Authors:** Edwina McCarthy, Danielle Mazza

**Affiliations:** 1 Department of General Practice School of Primary and Allied Health Care Monash University Melbourne Australia

**Keywords:** social media, Facebook, recruitment, intervention study, patient education, internet

## Abstract

**Background:**

Social media is a popular and convenient method for communicating on the Web. The most commonly used social networking website, Facebook, is increasingly being used as a tool for recruiting research participants because of its large user base and its ability to target advertisements on the basis of Facebook users’ information.

**Objective:**

We evaluated the cost and effectiveness of using Facebook to recruit young women into a Web-based intervention study (PREFER). The PREFER study aimed to determine whether an educational video could increase preference for and uptake of long-acting reversible contraception (LARC).

**Methods:**

We placed an advertisement on Facebook over a 19-day period from December 2017 to January 2018, inviting 16- to 25-year-old women from Australia to participate in a Web-based study about contraception. Those who clicked on the advertisement were directed to project information, and their eligibility was determined by using a screening survey.

**Results:**

Our Facebook advertisement delivered 130,129 impressions, resulting in over 2000 clicks at an overall cost of Aus $918 (Aus $0.44 per click). Web-based project information was accessed by 493 women. Of these, 462 women completed the screening survey, and 437 (437/463, 95%) women were eligible. A total of 322 young women participated in Surveys 1 and 2 (74% response rate), and 284 women participated in Survey 3 (88% retention rate), with an advertising cost of Aus $2.85 per consenting participant.

**Conclusions:**

Facebook proved to be a quick, effective, and cost-efficient tool for recruiting young Australian women into a study that was investigating contraceptive preferences. However, Web-based recruitment may result in sociodemographic biases. Further research is required to evaluate whether Facebook is suitable for recruiting older study populations.

## Introduction

Participant recruitment for research is challenging. This process involves identifying potentially eligible individuals, implementing strategies to target potential participants, inviting participants into the study, and obtaining informed consent. Traditional recruitment strategies include flyers, newspaper advertisements, mail-outs, word of mouth, and television broadcasts. However, these methods are often slow, labor intensive, and expensive, and these can lead to project delays, and, in some cases, failure to meet recruitment targets. Furthermore, difficulties sourcing participants and their contact details, the need to involve third-party organizations, the costs of staffing, travel, and printing, and the delay between obtaining consent and participation add further complexity.

Social media is a popular and convenient platform for communicating on the Web. Social media enables users to share information, such as updates, images, videos, and events, as well as send messages and maintain contact with other users. Facebook is the most commonly used social networking website, with over 2 billion users worldwide [1]. This includes 15 million users in Australia, with 50% of the Australian population using Facebook daily [2]. In Australia, over 2.5 million Facebook users are aged 18 to 25 years [2]. Facebook is increasingly being used as a tool for recruiting research participants. Its large user base and its ability to target advertisements according to demographic information made available by Facebook users make Facebook an effective recruitment approach. Facebook has been used successfully to recruit participants for studies on potentially sensitive topics, such as smoking cessation [3], alcohol consumption [4], abortion [5], and sexual health/HIV [6]. Facebook has also been successfully used to recruit participants for intervention studies on depression [7], posttraumatic stress disorder [8], physical activity [9], and smoking [10]. An Australian study that examined contraceptive use (CUPID) successfully used Facebook, as well as traditional methods, to recruit young women [11].

Although the use of Facebook as a recruitment technique is gaining popularity, the ability of researchers to draw upon the experiences of previous work is compromised by inconsistent reporting of Facebook recruitment. Thornton et al’s [12] systematic review of studies that used Facebook to recruit participants found that only half of the articles described their Facebook recruitment strategy in detail. Comparing Facebook recruitment outcomes is further complicated by the use of different measures to assess effectiveness (eg, impressions and cost per click). Although the cost per participant is generally a comparable measure across different methods, few studies report on recruitment costs [13]. There is also limited information on the development of advertising content used in Facebook recruitment and the role of incentives with online recruitment.

We used Facebook advertising exclusively to target young Australian women and recruit them into a Web-based intervention study, the PREFER study. The PREFER study was concerned with examining whether an educational video could increase young women’s preference for and uptake of LARC. This paper aimed to focus on our evaluation of the cost and effectiveness of using Facebook to recruit study participants.

## Methods

### Study Design

PREFER used a before-and-after survey methodology to address its aim. Participation in the study involved completing a series of Web-based surveys: (1) screening, (2) preintervention (Survey 1), (3) immediately postintervention (Survey 2), and (4) 6 months postintervention (Survey 3). This study was approved by the Monash University Human Research Ethics Committee (project number 10456).

### Recruitment

We created a paid Facebook advertisement, featuring an image of young people using computer devices and a call for volunteers to participate in Web-based surveys about their contraceptive preferences. The Facebook Ads Manager fed the advertisement into newsfeeds, targeting women aged 16 to 25 years, who were living in Australia.

Women were eligible to participate in the survey if they (1) were aged between 16 and 25 years, (2) had been sexually active with a male partner in the past 6 months or anticipated sexual activity in the next 6 months, (3) had not undergone a tubal ligation or hysterectomy, (4) had a partner or partners who had not undergone a vasectomy, and (5) were not pregnant or had no desire to become pregnant in the next year.

Power and sample size estimations determined that 281 participants would be required in this study. The budget for the recruitment of participants was Aus $2000.

### Data Collection

Facebook users who clicked on the advertisement were then taken to a project landing page via the Department of General Practice, Monash University website. The page outlined the phases of the study and included a link to the project’s explanatory statement. Interested individuals were then able to access the Web-based screening survey and confirm that they had read and understood the explanatory statement. Eligible participants could then access the baseline survey (Survey 1), and the completion and submission of Web-based survey responses were considered implied consent. On completion of the baseline survey, participants were directed to view the long-acting reversible contraception (LARC) first Web-based patient education video on the Web (approximately 10 min long), which conveyed information on all contraceptive options available to Australian women. This included their mode of action, effectiveness, and side effects, starting with discussion of the LARC options with emphasis on their superior efficacy and patient acceptability. Following the video (intervention), participants were directed to complete Survey 2. A total of 6 months later, participants were emailed a Web-based link to the postintervention survey (Survey 3). Participants received up to Aus $40 in electronic gift vouchers as reimbursement for their time—Aus $20 for completing Surveys 1 and 2 and Aus $20 for completing Survey 3.

### Data Analysis

We used the measures outlined in the following section to evaluate the cost and effectiveness of using Facebook to recruit female participants aged 16 to 25 years into the PREFER study.

#### Measures

Textboxes 1 and 2 show effectiveness and cost, respectively.

**Effectiveness measures.**

- Impressions: The number of times the Facebook advertisement appeared on the Newsfeeds of Facebook users.
- Clicks: The number of times Facebook users clicked on the advertisement.
- Number of completed screening surveys.
- Eligibility: The proportion of interested individuals who were eligible to participate.
- Participants: The number of participants who completed Surveys 1 and 2.
- Retention rate: The proportion of participants who completed Surveys 1 and 2, who completed Survey 3.

Timeframe: Surveys 1 and 2 completed by participants within 5 months.

**Cost measures.**

- Cost per click: The cost paid each time the advertisement was clicked during the advertising campaign.
- Cost per participant: The cost per consenting participant.
- Facebook advertising budget: Aus $2000 (excluding incentive payments of Aus $40 electronic gift card per participant).

## Results

### Overview

Our Facebook advertisement delivered 130,129 impressions, resulting in over 2000 clicks at an overall cost of Aus $918 (Aus $0.44/click). Web-based project information was accessed by 493 women. Of these, 462 women completed the screening survey and 437 (95%) women were eligible. This occurred in only 19 days. A total of 322 young women participated in Surveys 1 and 2 (74% response rate), and 284 women participated in Survey 3 (88% retention rate), with an advertising cost of Aus $2.85 per consenting participant. Recruitment outcomes are further described below.

### Recruitment Outcomes

Textboxes 3 and 4 show effectiveness and cost, respectively.

**Effectiveness measures.**

- Impressions: 130,129
- Clicks: 2101
- Completed screening survey: 462
- Eligibility: 95% (n=437)
- Participants: n=322
- Retention rate: 88% (n=284)

Timeframe: 19 days

**Cost measures.**

- Cost per click: Aus $0.44
- Cost per participant: Aus $2.85
- Total Facebook advertising cost: Aus $918

## Discussion

### Principal Findings

Our study showed that Facebook advertising was a cost-effective method for recruiting participants into an educational intervention study delivered on the Web. Our project budget of Aus $2000 for a 5-month recruitment timeframe was met with an overall spend of Aus $918 over a brief 19-day Facebook advertising campaign. We recruited 322 young Australian women, of whom 284 (88%) women completed the follow-up survey (Survey 3). These results add to the evidence that Facebook is a useful tool for recruiting research participants [14,15].

Our study is novel in that we used a single paid Facebook advertisement to successfully recruit young women. Facebook recruitment proved to be an effective strategy for recruiting participants who were eligible. Our Facebook advertisement targeted users by age (16-25 years), sex (female), and location (Australia), and the screening survey was completed by 462 people. Importantly, this method captured individuals who were eligible and willing to participate (n=437; 95%), with the remaining 5% ineligible because of not meeting the inclusion criteria. This confirms the findings of previous studies that showed that Facebook is effective in targeting and recruiting young Australian women [11,14,16]. However, unlike these studies, we were able to meet and exceed our recruitment target without combining Facebook recruitment with traditional recruitment methods or using multiple Facebook advertisements.

A surprising finding was that we achieved our participant target (n=281) within a 19-day timeframe. Owing to the cost-effectiveness of our recruitment campaign, we were able to increase our participant target from 281 to 320. Whitaker’s [15] review showed that it took an average of 5 months (median 3 months, IQR 8) to recruit 463 participants (median 264 participants, IQR 775). Our study had anticipated a 5-month timeframe for the recruitment of participants for Surveys 1 and 2; however, recruitment was completed within 19 days. Over this period, our advertisement appeared in Facebook newsfeeds more than 130,000 times. Despite reports of poor participant retention in other internet-based studies [17], participant retention was high at our 6-month follow-up (88% completed Survey 3). This highlights the potential strengths of recruiting young women for research by using Facebook and conducting follow-up via email. Using the internet to connect with and communicate with research participants enables them respond and complete Web-based surveys at a time and place convenient for them.

Facebook recruitment was also an affordable approach for recruiting participants into our study. We used less than half (Aus $918) of our recruitment budget (Aus $2000), and the cost per participant was only Aus $2.85 (US $2.05). Traditional recruitment methods have been estimated to cost US $1094.27 for television, US $811.99 for print media, US $635.92 for radio, and US $332.46 for postal recruitment [18]. Previous studies resorted to combining Facebook advertising with traditional recruitment methods such as mail-outs, radio promotion, newspaper advertising, and word of mouth [11,19,20] to achieve recruitment targets. Other studies combined Facebook advertising with other Web-based methods, such as Google, Web-based newsletters and email [3,20], or other social media platforms, such as Twitter [6] and MySpace [21]. Our streamlined recruitment approach of developing 1 Facebook advertisement enabled us to meet and exceed our original recruitment target, without the need to apply other recruitment strategies or develop alternative Web-based advertising content as required by other studies [11,14]. This facilitated our ability to track the interest in our advertisement over the course of our recruitment campaign.

Our cost per participant was also inexpensive compared with other studies that used Facebook advertising for recruitment. A systematic review of studies that used Facebook to recruit participants for health research found that the average cost per click was US $0.51 [14], compared with our study’s cost per click of US $0.32. Furthermore, our cost per participant was considerably less expensive than other studies that used Facebook to recruit young Australian women [11,14]. Our findings add to the growing evidence of the cost benefits of using Facebook to recruit research participants [12,15].

### Limitations

A limitation to our study is that did not collect reasons for nonparticipation because of the quick progress of participant recruitment and data collection. Reasons for nonparticipation may have been particularly useful to know if our recruitment target was not met, as it can assist researchers to direct their resources more appropriately to help progress recruitment efforts [11].

### Conclusions

For the purposes of recruiting young Australian women to our Web-based intervention, Facebook proved to be an affordable, effective, and quick method of recruiting participants. Our study adds important data on the outcomes of using Facebook to recruit research participants, and the study highlights the importance of documenting successful recruitment campaigns, particularly for sexual health research, which can be considered a sensitive topic. Our findings will inform researchers of the benefits of using Facebook to recruit participants; however, further research is required to establish the effectiveness of using Facebook to target other study populations, such as people in older age groups or from diverse cultural backgrounds.

## Abbreviations

LARC: long-acting reversible contraception
